# The Salt Swap intervention to reduce salt intake in people with high blood pressure: protocol for a feasibility randomised controlled trial

**DOI:** 10.1186/s13063-019-3691-y

**Published:** 2019-10-11

**Authors:** Sarah Payne Riches, Carmen Piernas, Paul Aveyard, James P. Sheppard, Mike Rayner, Susan A. Jebb

**Affiliations:** 1 Nuffield Department of Primary Care Health Sciences, University of Oxford Radcliffe Observatory Quarter, Woodstock Road, Oxford, OX2 6GG UK; 2 Nuffield Department of Population Health, University of Oxford, Richard Doll Building, Old Road Campus, Roosevelt Drive, Headington, Oxford, OX3 7LF UK

**Keywords:** Salt intake, Urinary sodium, Blood pressure, Food purchasing, Behaviour change

## Abstract

**Background:**

High salt intake is a risk factor for hypertension and cardiovascular disease. Reducing salt intake has been shown to reduce blood pressure. Despite population-level interventions, including product reformulation and public awareness campaigns, adult salt consumption in the UK still exceeds recommendations; this is primarily due to salt consumed in processed and pre-packaged foods. Moderate or high-intensity dietary advice to encourage individuals to reduce their salt intake has been shown to be effective at reducing blood pressure, but evidence of the effectiveness of interventions that are suitable for delivery at scale in routine primary care is scarce. This feasibility trial investigates a complex behavioural change intervention to reduce dietary salt intake and blood pressure by encouraging individuals to purchase lower-salt foods when grocery shopping.

**Methods:**

This randomised controlled trial will test the feasibility of a novel intervention to reduce salt intake, and the trial procedures to assess its effectiveness. We will recruit participants through UK general practices and randomise 40 participants with high blood pressure, in a 2:1 allocation to receive either the Salt Swap intervention or a control information leaflet. The primary outcomes relate to the criteria for progression to a large-scale trial. These include follow-up rates at 6 weeks, fidelity of intervention delivery and use of the intervention mobile app. Secondary outcomes include the effect of the intervention on the salt content of purchased foods (grams per 100 g), urinary sodium excretion assessed through 24-hour urine samples and blood pressure. Trial process measures will be collected and qualitative assessment will provide insights into participant engagement with the intervention content and perceived barriers to and facilitators of salt reduction dietary behavioural change.

**Discussion:**

If the outcomes indicate the trial is feasible and there is evidence that behavioural change may result in salt reduction, we will proceed to a definitive trial to test the effectiveness of the intervention to lower blood pressure. If successful, this intervention approach could be applied not only to people with high blood pressure, but also to the wider population with normal blood pressure in whom dietary salt intake exceeds recommendations.

**Trial registration:**

ISRCTN, 20910962. Registered on 5 April 2017.

## Background

Cardiovascular disease (CVD) is the leading cause of premature mortality globally and a significant contributor to preventable morbidity [1–4]. Hypertension is one of the most significant risk factors for CVD, and a key contributor to the global burden of disease [5, 6]. At least one quarter of adults in the UK (and more than half of those older than 60 years) have hypertension [7]. There is a positive association between blood pressure and dietary salt intake [8], both in individuals with hypertension and also in those with normal blood pressure [9]. Despite UK government recommendations for adults to reduce dietary salt intake to < 6 g/day, intake remains high at an average of 8.1 g/day [10], with around three quarters of this derived from packaged or processed foods [11]. Overall, 70% of the adult population are estimated to have salt intake > 6 g/day [10].

Clinical trials have proven that dietary interventions with intensive support to reduce salt intake, sometimes involving the provision of low-salt foods, can significantly reduce systolic blood pressure, by up to 7 mmHg [12–14]. In addition, systematic reviews have demonstrated that advice-based interventions such as nutritional counselling or tailored education can be effective [15, 16], but the effectiveness of these interventions depends largely on intensity. Interventions most likely to be effective are those including moderate-to-high-intensity advice, involving multiple one-to-one or group counselling sessions [17]. The challenge is that such interventions are not feasible to deliver at scale by non-specialist staff without a significant expansion in the workforce and training. At present any dietary advice in routine primary care usually consists of non-tailored information to help patients maintain a low-salt diet [7]. There is a wide range of salt content in foods within and between product categories, and it is difficult for non-specialists to provide accurate and personalised advice to help individuals achieve a low-salt diet.

Brief behavioural interventions have been used successfully in primary care to encourage smoking cessation [18] and reductions in alcohol intake [19] but evidence on the effectiveness of brief interventions for dietary change is limited. A recent pilot study of a low-intensity dietary counselling intervention to reduce blood cholesterol demonstrated a significant beneficial short-term effect [20], indicating potential for a brief-intervention approach. However, a study of a brief intervention to reduce blood pressure, covering a range of strategies including diet, showed no effect [21], and there is currently no established brief intervention known to be effective to reduce salt intake.

One approach to magnify the effectiveness of advice-based interventions for dietary change is to use technology to deliver personalised advice at the point at which individuals make a choice about the food and drink they buy or consume. Studies using digital tools such as smartphone applications (apps) as a standalone intervention to reduce salt intake indicate their potential effectiveness [22, 23].

This study investigates the feasibility of a trial examining a complex, behaviour change intervention to reduce salt intake, combining brief advice delivered by non-specialist health practitioners in primary care with use of an app to guide food-purchasing decisions. It aims to motivate and support individuals with high blood pressure to reduce their salt intake by swapping to lower-salt products when grocery shopping. A definitive trial to show whether this intervention is effective at lowering salt intake and blood pressure would need to be large and would be expensive. Before this is justified, we need to ensure that people engage with the intervention, that it can reduce salt intake in the short term and that the research methods run as expected.

## Objectives

The primary aim of this study is to test the feasibility of a randomised controlled trial (RCT) to study the effectiveness of this complex behavioural intervention to lower salt intake in people with raised blood pressure. The primary objectives are therefore to assess the criteria for progression to a full trial, which would examine the effect on blood pressure. The primary objectives will assess the:

- Rate of attendance at the follow-up session
- Proportion of the essential intervention elements that are delivered in the intervention session
- Proportion of participants using the Salt Swap app

Secondary objectives are to:

- Gain an indication of effectiveness of the intervention in terms of salt intake, salt content of purchased foods and blood pressure at follow-up
- Understand the barriers and facilitators of successful engagement with the intervention

We will also test the trial procedures through a range of process measures.

## Methods

### Trial design and setting

This trial will be an individually randomised, parallel, two-arm, controlled feasibility trial with a 2:1 (intervention to control) allocation ratio. The primary outcome will be the criteria to progress to a full trial. Process evaluation and analysis of effectiveness outcomes may inform a future trial. Participants will be recruited through five primary care practices in Oxfordshire, UK.

### Recruitment

Practices will search the electronic health records of registered patients to find people who meet the age and blood pressure inclusion criteria. Prior to sending out the invitation letters, the general practitioner (GP) will screen the search results to ensure that all those identified are medically appropriate to participate in the trial and meet the clinical inclusion criteria. The practice will send potential participants an invitation letter asking them to contact the research team if they are interested in participating; the letter will also include a link to an electronic version of the participant information sheet. GPs may also identify eligible patients opportunistically during routine consultations or via the National Health Service (NHS) Health Check programme. We will aim to randomise 40 participants in the study.

### Eligibility criteria

#### Inclusion criteria

The inclusion criteria are:

- Most recent systolic blood pressure reading in the past 2 years > 130 mmHg if they are currently taking anti-hypertensive medication or > 140 mmHg if non-medicated
- If on pharmacological treatment for hypertension, the participant has been prescribed a stable dose for at least 6 weeks
- Willing and able to give informed consent for participation in the study
- Male or female, aged between 18 and 80 years
- English speaking
- Regularly shop in a supermarket (excluding online supermarkets), spending at least £25 at least once a fortnight
- Own a smartphone (android or iOS) and prepared to use an app for healthy eating
- Express a desire for support to improve the nutritional quality of their diet to reduce their CVD risk

#### Exclusion criteria

The exclusion criteria are:

- Currently on a clinician-supervised diet or a restricted diet
- Currently using or have experience of using the Foodswitch or Saltswitch apps
- Unable to read and understand the instructions provided in English
- Have secondary, previous accelerated or malignant hypertension documented in the electronic patient record
- Currently being assessed for diagnosis of hypertension
- Currently on any medication that may lead to hyponatraemia or fluid retention
- Existing or recent cardiovascular conditions: heart attack or stroke within the last 3 months, heart failure of New York Heart Association grade II and more severe or prolonged QT syndrome, angina, arrhythmia or atrial fibrillation
- Currently participating in another research study
- Considered by the GP as not suitable for or unable to meet the demands of the study or unlikely to comply with study procedures as stated in the protocol
- Planning to be away from home for more than 2 weeks consecutively during the 6-week intervention period

### Intervention and comparator

#### Intervention

This study investigates the Salt Swap intervention, which aims to help people swap to lower-salt alternatives for everyday foods when grocery shopping. The intervention development was informed by two established behaviour change frameworks, the Behaviour Change Wheel [24] and the Theoretical Domains Framework (TDF) [25]. These theoretical frameworks were used to identify the key determinants of behaviour, particularly the aspects of capability, opportunity and motivation, that need to be influenced in order to support people to reduce purchases of high-salt foods and drinks. The intervention development is described elsewhere.

The intervention incorporates motivation and education, problem solving, goal setting and action planning. It comprises two main components: brief advice provided by a healthcare professional (HCP) at the GP practice and instructions to use the Salt Swap app. Healthcare professionals, typically practice nurses or healthcare assistants, will be trained by one face-to-face training session with a researcher and by video-based training materials. The content of the 20-min one-to-one advice session is based on best practice guidance and information by expert sources including NHS Choices and the British Heart Foundation (BHF) [26, 27]. It is supported by the Salt Swap booklet given to participants during the intervention visit.

This advice incorporates information on:

- The impact of salt on health and blood pressure in particular
- The health benefits of salt reduction
- Recommended salt intake
- Advice for cutting salt intake
- Examples of lower-salt alternatives
- Salt reduction goal setting
- Self-assessment
- Action planning to reduce salt intake

**Fig. 1:**
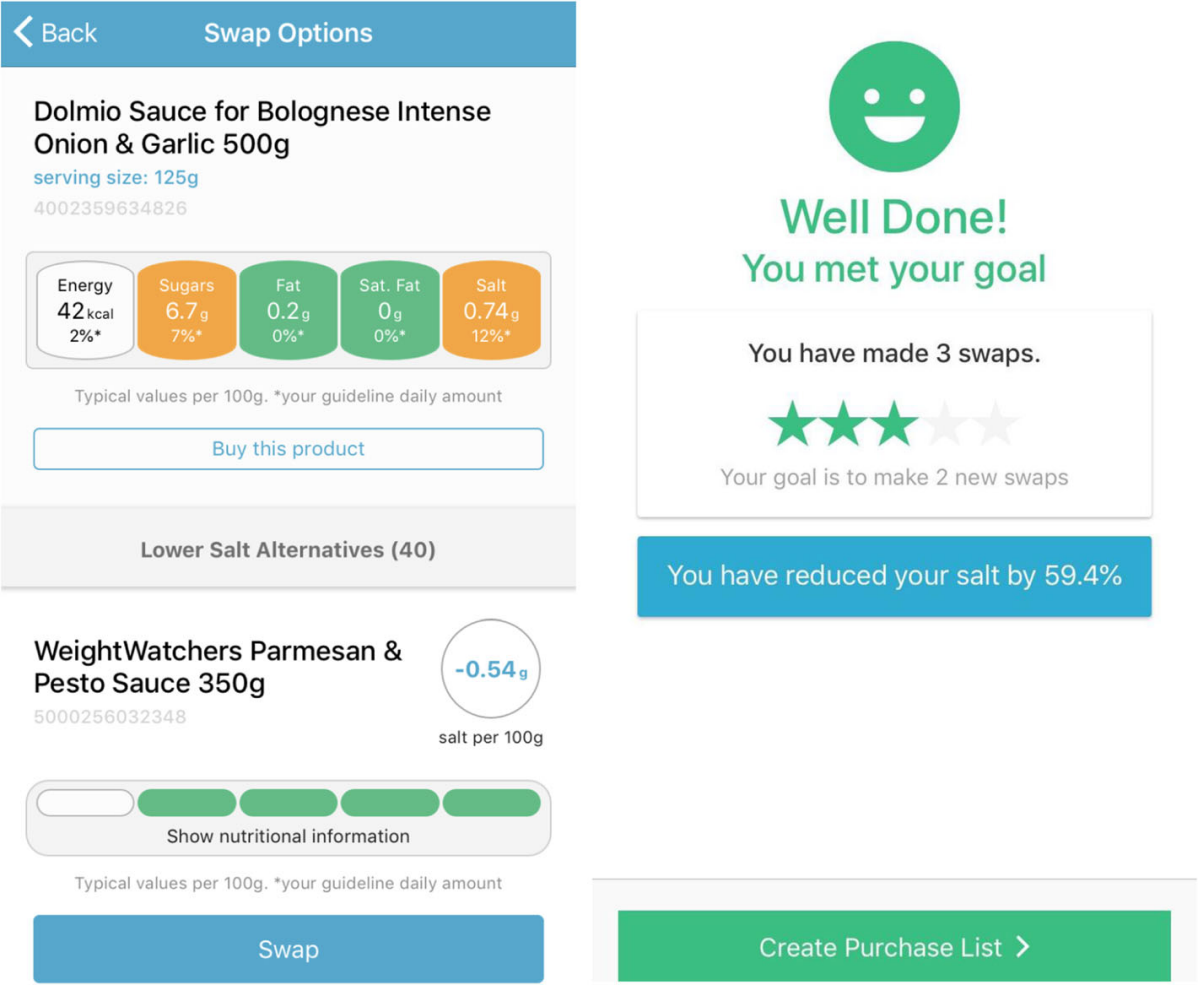
Salt Swap app

In addition, during this session participants are encouraged to download a novel mobile phone app, Salt Swap, which delivers tailored, salient information on the salt content of products. Users scan a product barcode to receive nutritional information, presented using the traffic light labelling system recommend for use in the UK [28], and are shown suggested lower-salt alternatives. The app contains a database of food and drinks available in UK supermarkets and their nutritional composition. The app allows users to set goals for salt reduction and to receive feedback on the achievement of these goals and the salt reduction achieved through swapping to lower-salt products (see Fig. 1). All other clinical care will continue as usual.

#### Control (usual care)

Participants randomised to the control arm will receive a copy of the publicly available BHF *Cut Down on Salt* booklet [27] by post, or any revised version of this publication. On completion of their follow-up visit, i.e. when they have completed their participation in the study, they will be sent the same written advice that is given to intervention participants. All other clinical care will continue as usual.

### Participant flow

**Fig. 2:**
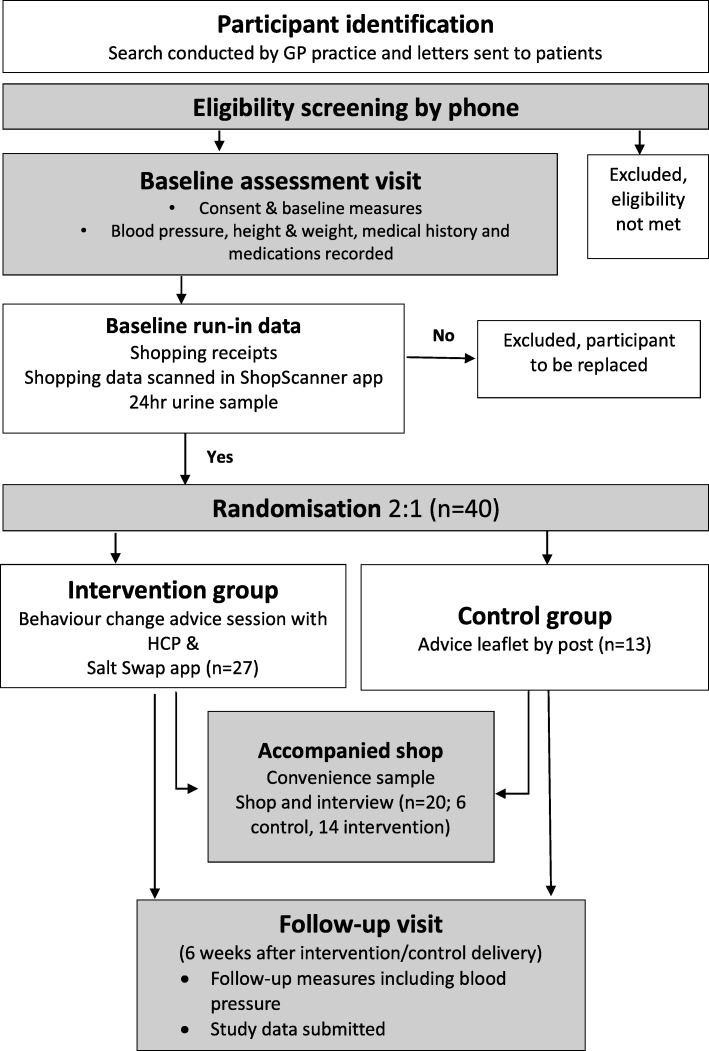
Participant flow. GP, general practitioner; HCP, healthcare professional

Figure 2 illustrates the trial flowchart.

#### Screening and informed consent

Participants who are interested in taking part will contact the research team. A researcher will assess eligibility based on the full inclusion and exclusion criteria, verbally provide information as described in the participant information sheet and answer any questions about participation in the study. Those who are eligible will be booked into an initial face-to-face baseline study visit at their GP practice. When the participant attends the baseline visit, the researcher will provide verbal and written information about the trial and seek informed consent (see Table 1 for the schedule of procedures). Having had a chance to ask questions, those individuals willing to participate will give written informed consent by means of a participant-dated signature. The consent form will also be dated and signed by the researcher who presented and obtained the informed consent.

**Table 1:** Schedule of study visits, assessment and procedures

	Screening phone call	Enrolment and baseline (Visit 1)	Allocation	Intervention (Visit 2)	Accompanied shop (Visit 3)	Followup (Visit 4)
Time point (weeks from baseline)	−1	0	2	2–3	4–9	9–10
Responsibility	Central research team	Central research team	Independent researcher	Healthcare professional	Central research team	Central research team
STUDY PROCEDURES						
Eligibility assessment	✓					
Informed consent		✓				
Baseline assessment including:		✓				
Blood pressure		✓				
Height and weight		✓				
Medical history and medications		✓				
Shopping and diet questionnaire		✓				
Instruction on baseline run-in data collection		✓				
Urine sample collection		✓ (+ up to 2 weeks)				
Randomisation			✓			
Intervention delivery				✓		
Accompanied shop and interview					✓	
Follow-up outcome measures including:						✓
Blood pressure						✓
Medical history and medication review						✓
Intervention acceptability questionnaire						✓
Urine sample collection						✓ ✓ (+ up to 7 days)

#### Baseline

The baseline assessment will be undertaken at the initial baseline visit following informed consent. The researcher will collect clinical measures of height (metres), weight (kilograms), and blood pressure (seated systolic and diastolic blood pressure, measured after 5 min rest and using the mean of the second and third of three readings taken 1 min apart using a validated digital automatic blood pressure monitor, the OMRON M10-IT). The researcher will also record demographic information, relevant medication history and medications (verified through the patient’s electronic clinical record) and details of the participant’s shopping behaviour including the frequency of shopping, the number of people for whom they are shopping, the types of stores or settings most commonly visited for groceries and key influences on purchasing decisions (using an investigator-designed questionnaire).

At the end of the baseline visit participants will be instructed on how to use a mobile phone app to record their grocery shopping throughout the study. The app, called ShopScanner, is linked to a food and nutrient information database to enable recording of the nutrient content of purchased foods. They will also be asked to collect their grocery shopping receipts throughout the study.

#### Baseline run-in period

The baseline period will run for 2 weeks following the baseline assessment visit. Participants will be asked to collect a 24-hour urine sample within this baseline period, for the measurement of urinary sodium excretion (mmol/24-hour). They will also be asked to submit their grocery shopping till receipts for this period to the research team. Participants who return partial baseline data will be prompted, by phone or email, to return all requested data.

Participants will be randomised once the urine sample and baseline shopping data for the baseline period have been provided. Participants who do not complete these measures will not be randomised. Participants randomised to the intervention group will be booked to attend an intervention session with a HCP at their GP practice. Control participants will receive the control intervention leaflet by post.

#### Intervention visit

The intervention will be delivered by a HCP at the GP surgery. Designated HCPs from the surgery will be specifically trained to deliver the intervention and to complete an intervention checklist. This visit will last 20–30 min.

#### Follow-up

All participants will be invited to attend one follow-up assessment 6 weeks after they receive their allocated intervention (either the intervention visit or the control information sent by post). During this visit, a researcher will measure blood pressure and document any changes to relevant medical history or medications. Participants will be asked to complete an investigator-designed questionnaire to assess aspects of the intervention (or control leaflet) they received.

The follow-up questionnaire will collect data on changes in knowledge about dietary salt intake, awareness of salt intake and dietary-salt-related behaviours. Separate questions for each group will assess how helpful the Salt Swap intervention (advice session and Salt Swap app) or control leaflet were (1–5 scale from very unhelpful to very helpful) as well as the specific reasons why it was considered helpful or unhelpful (for the Salt Swap intervention only). The intervention group questionnaire will also collect data on app acceptability (“would you be interested in using a free app like the Salt Swap app if it was publically available in future?”) and frequency of use (always, most of the time, occasionally, never). Both groups will be asked if any other forms of support (e.g. apps, tools, advice or contact with health professionals) were used throughout the study.

Participants will be asked to submit their grocery shopping receipts for the past 6 weeks and to provide a second 24-hour urine sample at this appointment or within a week of their follow-up visit. The research team will be responsible for arranging follow-up visits. Participants will be contacted by phone in the first instance (unless an alternative contact method is requested by the participant). The research team will use text messages to remind participants of their upcoming appointments.

#### Accompanied shop

We will undertake an accompanied grocery shopping trip with a convenience sample of participants (both intervention and control), using the think-aloud method. The aim of this is to understand how the study materials and content influence shopping and wider dietary behaviours, and to assess the barriers to and facilitators of using the Salt Swap app.

Retrospective questioning via a semi-structured interview immediately after the accompanied shop will add depth to the information gathered about the participants’ thought processes and understanding of the role of diet in blood pressure control. This qualitative assessment will be undertaken at staggered time points throughout the study.

### Randomisation

Once participants have provided consent and completed the baseline data collection, the research team will randomise them to one of the two trial arms using computer-generated randomisation. The randomisation will be stratified by general practice using block randomisation in blocks of six. A researcher not involved in taking consent or measuring study outcomes will generate the random number sequence using an online random sequence generator (www.sealedenvelope.com) and assign participants to the intervention groups according to the allocation sequence, to ensure allocation is concealed from the rest of the research team until interventions are assigned. Both the research team and participants will be unaware of the treatment allocation prior to consent.

### Blinding

It is not possible to blind the participants, the clinicians delivering the intervention or the research team to treatment group due to the nature of the intervention. The main trial outcomes are progression criteria and process measures, which are unlikely to be influenced by knowledge of treatment allocation. Intervention effectiveness measures comparing trial arms, which would be used as the primary outcome in a future RCT, are objectively measured outcomes and not open to interpretation by researchers.

### Primary outcomes

The primary objective of the trial is to determine whether to progress to a full trial or whether substantial amendments to the trial procedures are required. Based on previous trials of a similar nature we would hope to achieve 80% for the first two progression criteria. Allowing for the 95% confidence interval around this point estimate, we have set the minimum criteria for immediate progression at 65%.

Level one: the following criteria must be met to progress to a full trial with minimal amendments to trial procedures:

1. That 65% of participants attend the follow-up session.
2. Healthcare professionals typically conduct a brief advice session where at least four out of six of the essential intervention elements are present, assessed by comparing the delivered intervention against a checklist of the components required by the protocol, using audio recordings of the consultation.
3. At least 50% of participants use the app to scan products on at least one occasion by the end of month one.

Level two: the following criteria must be met to continue to a full trial with substantial amendments to trial procedures:

1. At least 40% of participants attend the follow-up session.
2. Healthcare professionals typically conduct a brief advice session where at least a mean of three out of six of the essential elements are present.
3. At least 40% of participants use the app to scan products on at least one occasion by the end of month one.

If level-two criteria are not met, it would not be considered feasible to continue to a full trial with this trial design.

### Secondary outcomes and process measures

The secondary outcomes include both effectiveness and process measures. The trial is not powered to detect significant differences in effectiveness outcomes. A mix of quantitative and qualitative measures will be used to undertake a process evaluation and to inform sample size estimates for a future trial.

#### Secondary outcomes (effectiveness)

The secondary outcomes will be:

- Change in salt content of food purchases in the shopping basket, measured as salt in grams per 100 g (and in grams per megajoule) in the intervention compared to the control group; assessed throughout the study through collection of the nutrient composition of all purchased products.
- Change in salt intake measured through urinary sodium analysis in the intervention compared to the control group; assessed using 24-h urine collection during the baseline period and at the end of the 6-week follow-up appointment. Sodium excretion, reported as millimoles per litre, millimoles per 24 h and total volume of the urine sample, will be converted to daily salt intake in grams using the equation: 17.1 mmol of sodium = 1 g salt, and assumes all of the sodium was derived from salt.
- Change in blood pressure (systolic and diastolic) in the intervention compared to the control group; recorded at baseline and at the 6-week follow-up.

#### Process measures

The process measures will be:

- Recruitment rates: total recruited and number per practice per month, (including the number invited who were eligible and consented, and the number of recruited participants that completed all baseline assessments and were randomised).
- Acceptability of the intervention delivery. The perspective of the healthcare professionals responsible for delivering the intervention will be captured via interviews, exploring barriers to and facilitators of delivery and their confidence and belief in their capability to deliver the intervention session.
- App use and acceptability; measured quantitatively through within-app automatic recording (e.g. frequency of use, number of products scanned per user, frequency of swaps presented and accepted, use of specific behaviour change functionality such as goal setting), and measured qualitatively through the post-intervention survey and think-aloud accompanied shopping trips.
- Feasibility of data collection: willingness of participants to submit 24-hour urine samples and completeness of shopping data (including completeness of data on the salt content of purchased foods).
- Evidence of contamination; measured as use of the Salt Swap app by the control group.
- Changes in participant knowledge, perceptions and behaviours in relation to salt intake and health, measured qualitatively through participant questionnaires and semi-structured interviews.

### Sample size

This study is a feasibility study and is not powered to detect a significant intervention effect. However, we calculated that a sample size of 40 was sufficient to estimate our progression criteria outcomes within acceptably narrow confidence intervals to enable robust testing of the trial methods. Recruiting 40 patients will enable the anticipated follow-up rate of 80% to be estimated within +/− 12% (80%, 95% C.I. 68–92).

### Planned analysis

Baseline characteristics will be reported by trial arm using summary descriptive statistics.

#### Progression criteria

Descriptive statistics with 95% confidence intervals will be reported. Progression criteria will use data from all randomised participants.

#### Effectiveness measures

The analysis will test the change in urinary sodium and change in blood pressure. These effectiveness outcomes will be analysed on an intention-to-treat basis, using regression with baseline adjustment to calculate difference in means, and we will present these summary statistics with 95% confidence intervals. We will test for normality of the residuals and use the non-parametric Mann-Whitney U test, if this model is unacceptable. The analysis will test the change in salt content of food purchases from baseline over the 6-week follow-up, using linear mixed-effects regression models to account for repeat observations. Effectiveness analysis will be based on data from all participants for whom both a baseline and a follow-up measure is available. We will examine the sensitivity of the results to confounding due to differences in baseline characteristics. We will also adjust for the number of people in the household.

#### Process measures

Descriptive statistics with 95% confidence intervals will be used to report the process measures. Process measures will be based on all data available, regardless of whether participants completed the trial or withdrew. We propose to use framework analysis, a thematic research method, to analyse the data from the think-aloud shopping sessions and retrospective interviews [29, 30].

### Adverse events

We will only provide standard dietary advice consistent with recommendations from scientific advisory committees in the UK and internationally and we do not expect any related adverse events. However, any related, unexpected, serious adverse events (RUSAEs) will be recorded and reported to the Research Ethics Committee in accordance with the Health Research Authority (HRA) process. The duration of the serious adverse event (SAE) recording period lasts from enrolment in the study to the end of the termination of their programme. All RUSAEs will be recorded at the time the research team become aware of the incident.

An SAE is defined as any untoward medical occurrence that:

- Results in death
- Is life-threatening
- Requires inpatient hospitalisation or prolongation of existing hospitalisation
- Results in persistent or significant disability/incapacity
- Consists of a congenital anomaly or birth defect

All study-related SAEs will be included in the final trial report.

### Data management

The trial will be run in accordance with Good Clinical Practice (GCP) and access to data will be restricted to appropriately trained members of the research team and host organisation as necessary for audit and regulatory purposes. Data will be kept in accordance with the Data Protection Act, 1998 (DPA). Participant-identifiable information will be available to the researcher responsible for conducting accompanied grocery shopping visits and follow-up visits, as it is important that these data are known to the researcher. Otherwise, confidentiality will be maintained.

Data will be entered by a trained researcher directly into a secure online database incorporating an electronic case report form (CRF), using a unique participant ID. The database includes data validation checks and, prior to database lock, a dataset review will be undertaken by the chief investigator to ensure all queries have been resolved and the dataset is complete. All participant-identifiable data linked to the participant via their unique ID will be kept separately from anonymised research data in a password-protected file. Audio recordings will only be stored with participant ID in a password-protected file on secure University of Oxford servers. A study-specific data management plan (DMP) will be in place detailing the procedures necessary to ensure that high-quality data are produced for statistical analysis.

### Trial management committee

The Trial Management Committee will comprise the authors of this protocol, along with two members of the public. The chief investigator will be responsible for project coordination, with oversight from the senior co-investigators. All investigators will be trained in GCP, and will take appropriate actions to safeguard participants and the quality of the trial.

## Dissemination

The results of this research will be published in academic peer-reviewed journals and through presentations at relevant conferences. They will also be communicated to study participants and members of the public involved in the research through email newsletters and press releases targeted to the specific audience. Authors will acknowledge the study funders. Authorship will be determined in accordance with the International Committee of medical Journal Editors (ICMJE) guidelines and other contributors will be acknowledged.

## Discussion

This study will determine the feasibility of a novel, brief intervention to reduce salt intake, and of the trial design and methods to collect outcome measures. The growth of smartphone (and app) use provides an opportunity to develop behaviour change interventions that can reach large numbers of the population. There is an increasing body of evidence that interventions using apps to change dietary behaviour can be effective [31]. Furthermore, interventions encouraging people to swap to healthier products while grocery shopping in-store or online have also been shown to have potential for changing purchasing behaviour [32]. This study will add to the evidence base in this area by providing feedback on the acceptability of and engagement with interventions of this nature. It will address an important gap in the literature on how to provide interventions at scale to support people with high blood pressure to reduce their salt intake.

In the field of behaviour change, researchers are increasingly encouraged to report the evaluation of behaviour change interventions using established taxonomies and ontologies [33]. The outcomes included in this trial will show whether participants engage with the intervention as a whole and help us to understand their engagement with the specific behaviour change techniques incorporated within it. The think-aloud grocery shopping sessions and interviews with participants will provide real-world observations on the usability of the intervention. Furthermore, the qualitative and quantitative feedback from healthcare professions will enable us to assess the feasibility of delivering the intervention as part of routine care in primary care. A further strength of the study is the inclusion of clinical outcomes measures, which would be used in a future trial to evaluate clinical effectiveness.

This study utilises a novel method for collection of grocery shopping data, in addition to the more standard method of collecting till receipts. This has the potential for more efficient and robust assessment of nutrient outcomes, and the trial will provide valuable evidence on the feasibility of this method.

The study is limited in its ability to assess the study outcomes among a diverse range of individuals, due to the small sample size and regional recruitment area. Similarly, the small sample size limits the ability to assess the effectiveness of the intervention. The single follow-up assessment at 6 weeks is appropriate for a feasibility-stage trial but a future large-scale trial would include a longer-term follow-up in order to measure sustained effects on behaviour change and the impact on blood pressure. However, if the outcomes indicate the trial is feasible, and there is potential evidence of behavioural change, this study will provide valuable data to inform power calculations for a future trial on the effectiveness in reducing salt intake and lowering blood pressure in a broader study population.

If successful, this intervention approach could be applied not only to people with high blood pressure but also to the wider population with normal blood pressure in whom dietary salt intake exceeds recommendations.

## Trial status

Recruitment of participants began on 25 September 2018 and will be open until May 2019. The current trial protocol is version 2.0 dated 20 July 2018.

## Data Availability

The data are not publicly available as they contain information that could compromise research participant privacy and consent. The data that support the findings of this study are available on reasonable request from the corresponding author (SPR).

## Abbreviations

app: Smartphone application
BCW: Behaviour Change Wheel
CRF: Case report form
CVD: Cardiovascular disease
DMP: Data management plan
GCP: Good Clinical Practice
GP: General practitioner
HCP: Healthcare professional
HRA: Health Research Authority
ICMJE: International Committee of Medical Journal Editors
ISRCTN: International Standard Randomised Controlled Trials Number
NHS: National Health Service
NICE: National Institute for Health and Care Excellence
RCT: Randomised controlled trial
RUSAE: Related, unexpected, serious adverse event
REC: NHS National Research Ethics Committee
SAE: Serious adverse event
TDF: Theoretical domains framework
